# Human Papillomavirus Vaccine Efficacy and Effectiveness against Cancer

**DOI:** 10.3390/vaccines9121413

**Published:** 2021-11-30

**Authors:** Supitcha Kamolratanakul, Punnee Pitisuttithum

**Affiliations:** Vaccine Trial Centre, Faculty of Tropical Medicine, Mahidol University, 420/6 Ratchawithi Road, Ratchathewi, Bangkok 10400, Thailand; supitcha.kam@mahidol.edu

**Keywords:** human papillomavirus (HPV), HPV vaccine, cervical cancer, other HPV-related cancers, vaccine efficacy, vaccine effectiveness

## Abstract

Human papillomavirus (HPV) is the most common sexually transmitted infection, with 15 HPV types related to cervical, anal, oropharyngeal, penile, vulvar, and vaginal cancers. However, cervical cancer remains one of the most common cancers in women, especially in developing countries. Three HPV vaccines have been licensed: bivalent (Cervarix, GSK, Rixensart, Belgium), quadrivalent (Merck, Sharp & Dome (Merck & Co, Whitehouse Station, NJ, USA)), and nonavalent (Merck, Sharp & Dome (Merck & Co, Whitehouse Station, NJ, USA)). The current HPV vaccine recommendations apply to 9 years old and above through the age of 26 years and adults aged 27–45 years who might be at risk of new HPV infection and benefit from vaccination. The primary target population for HPV vaccination recommended by the WHO is girls aged 9–14 years, prior to their becoming sexually active, to undergo a two-dose schedule and girls ≥ 15 years of age, to undergo a three-dose schedule. Safety data for HPV vaccines have indicated that they are safe. The most common adverse side-effect was local symptoms. HPV vaccines are highly immunogenic. The efficacy and effectiveness of vaccines has been remarkably high among young women who were HPV seronegative before vaccination. Vaccine efficacy was lower among women regardless of HPV DNA when vaccinated and among adult women. Comparisons of the efficacy of bivalent, quadrivalent, and nonavalent vaccines against HPV 16/18 showed that they are similar. However, the nonavalent vaccine can provide additional protection against HPV 31/33/45/52/58. In a real-world setting, the notable decrease of HPV 6/11/16/18 among vaccinated women compared with unvaccinated women shows the vaccine to be highly effective. Moreover, the direct effect of the nonavalent vaccine with the cross-protection of bivalent and quadrivalent vaccines results in the reduction of HPV 6/11/16/18/31/33/45/52/58. HPV vaccination has been shown to provide herd protection as well. Two-dose HPV vaccine schedules showed no difference in seroconversion from three-dose schedules. However, the use of a single-dose HPV vaccination schedule remains controversial. For males, the quadrivalent HPV vaccine possibly reduces the incidence of external genital lesions and persistent infection with HPV 6/11/16/18. Evidence regarding the efficacy and risk of HPV vaccination and HIV infection remains limited. HPV vaccination has been shown to be highly effective against oral HPV type 16/18 infection, with a significant percentage of participants developing IgG antibodies in the oral fluid post vaccination. However, the vaccines’ effectiveness in reducing the incidence of and mortality rates from HPV-related head and neck cancers should be observed in the long term. In anal infections and anal intraepithelial neoplasia, the vaccines demonstrate high efficacy. While HPV vaccines are very effective, screening for related cancers, as per guidelines, is still recommended.

## 1. Introduction

Human papillomavirus (HPV), a DNA virus from the Papillomaviridae family, is one of the most common sexually transmitted agents. More than 40 human papillomavirus types can infect the genital areas of men and women, including the skin of the penis; the vulva (area outside the vagina); the anus; and the linings of the vagina, the cervix, and the rectum. These types can also infect the lining of the mouth and the throat. However, a total of 15 HPV genotypes are associated with the development of cervical cancer [1], and at least one of these types can cause cancers of the vulva, the vagina, the penis, and the anus and certain head and neck cancers (specifically, the oropharynx, which includes the back of the throat, the base of the tongue, and the tonsils) [2]. HPV 16 accounts for approximately 95% of HPV-positive oropharyngeal carcinomas.

Globally, decreasing trends were observed in the incidence, death, and disability-adjusted life years (DALYs) of cervical cancer from 1990 to 2019. The age-standardized death rate (ASDR) showed a decreasing trend with an annual average reduction of 0.93% (estimated annual percentage change (EAPC) = −0.93; 95% confidence interval (CI) = −0.98 to −0.88)) The ASDR showed a downward trend in all SDI areas, particularly high-SDI areas, such as Central and Latin America (EAPC = −1.57; 95% CI = −1.68 to −1.46). However cervical cancer is still one of the most common cancers in women. Cervical cancer cases increased by 6.5% in 2020. The distribution of cervical cancer varies throughout the world. The mortality rate is still high, especially in transitioned countries (12.4 per 100,000); the largest number of deaths was observed in South Asia (1833.69 × 10^3^) [3]. 

Cervical cancer incidence is related to age, with the highest incidence rates in the 50 to 54 age group; the mortality rate is high in older women (females aged 75 and over), suggesting infection at a younger age and slow progression to cancer. The 5-year survival rate for all people with cervical cancer is 66.3% [4]. Vaccines against HPV were introduced on the recommendation of the World Health Organization (WHO) and have been available since 2009, 2014, 2017, and 2019 [5]. The recommendations include that the primary target population for HPV vaccination should continue to be girls aged 9–14 years, prior to their becoming sexually active, to be administered two doses, and girls ≥ 15 years of age, including those younger than 15 years known to be immunocompromised and/or HIV-infected (regardless of whether they are receiving antiretroviral therapy), to be administered three doses. In the context of supply constraint, introduction of multiple age-cohorts vaccination, gender-neutral, and older-age-group vaccination strategies in any country should be temporarily postponed until all countries have been able to introduce HPV vaccination in at least one age-cohort (i.e., a single year each cohort) of the WHO-recommended primary target population of 9- to 14-year-old girls [6]. 

Recently, the report from a nationwide cohort study in Denmark showed an 86% decrease in cervical cancer among 16-year-old and younger people and a 68% decrease among older teens [7] and reported a non-statistically significant increase in cervical cancer among women vaccinated between the ages of 20 and 30 years compared with unvaccinated women. However, the Swedish study found a 62% decrease in cervical cancer among women vaccinated between 20 and 30 years of age [7,8]. The World Health Organization (WHO) has set a goal for the global elimination of cervical cancer, defined as an incidence of fewer than 4 per 100,000 women per year. A 90-70-90 target has been set: 90% of girls should be fully vaccinated with human papillomavirus (HPV) vaccine by age 15, 70% of women should be screened with a high-performance test by 35 and again by 45 years of age, and 90% of women with cervical disease should receive treatment [9]. The need for the target arises from the slow roll out of HPV vaccination, low levels of screening and early detection of cervical cancer, as well as limited access to comprehensive cancer treatment. Unfortunately, the major burden (86%) of cervical cancer is in low- and middle-income countries but <30% of these countries have introduced the vaccine. The major impediment is the cost, both of the vaccine and also of delivery to adolescents in countries that have limited infrastructure for adolescent immunization. The situation is exacerbated by constraints in vaccine supply, likely to persist until 2022/25. Alternative scheduling and/or reduction of doses are under discussion. The initially recommended three-dose schedule, priming doses at 0 and 1/2 months followed by a boost at 6 months, was changed in 2014 by WHO SAGE for adolescents at <15 years of age to two doses, at 0 and 6 or 12 months [10]. Evidence is accumulating from national immunization programs, post hoc analyses of the RCTs, and a large observational cohort study that one dose may be enough to provide protection for at least 7–10 years against persistent HPV infection and high-grade cervical disease [11]. The WHO 2030 interim targets in the global strategy on the elimination of cervical cancer includes updating the regional implementation guidance on the prevention and management of cervical cancers and strengthening health systems at all levels for the provision of cancer control services through a people-centered approach. It is necessary to include these services in the essential services packages toward universal health coverage to meet the global targets [12].

This review includes mostly HPV-related cervical cancers as well as other HPV-related diseases. However, to complete the picture of HPV vaccines for all cancers, a brief review on therapeutic HPV vaccines is also included.

## 2. Human Papillomavirus Vaccine

Mostly all cases of cervical cancer are a result of persistent infection with oncogenic HPV types. HPV vaccines protect against infection from human papillomaviruses (HPV). Three vaccines that prevent infection with disease-causing HPV have been licensed: bivalent (Cervarix), which prevents HPV 16 and 18 infection; quadrivalent (Gardasil), which prevents HPV types 6/11/16/18 infection; and nonavalent (Gardasil 9), which prevents HPV 6/11/16/18/31/33/45/52/58 infection.

All three vaccines are based on non-infectious recombinant type-specific L1 capsid proteins assembled into viral-like particles (VLPs) as immunogens. The expressed recombinant L1 capsids self-assemble, closely mimicking HPV virions, and it is this multiplicity of L1 domains that bestows the VLP antigen with high immunogenicity, even in the absence adjuvants [13].

In June 2006, the Advisory Committee on Immunization Practices (ACIP) recommended routine vaccination with HPV vaccine for females aged 11 or 12 years and catch-up vaccination for those aged 13 through 26 years. In 2011, a recommendation was made for routine vaccination of everyone through age 26 years. In 2019, ACIP recommended catch-up HPV vaccination for everyone through age 26 years, recognizing that some persons who are not adequately vaccinated might be at risk for new HPV infections and might benefit from vaccination in the age range of 27–45 years. 

WHO recommended HPV vaccine for girls from 9 years of age (female only). An HPV vaccine two-dose schedule is recommended for people who get the first dose before their 15th birthday and the interval between the two doses is to be 6–12 months. (0, 6- to 12-month schedule). A three-dose schedule is recommended for people who get the first dose on or after their 15th birthday and for people with certain immunocompromising conditions. (0, 1- to 2-month, 6-month schedule) [14].

### 2.1. Safety

The overall local adverse effects at the injection site were more common in vaccinated participants compared with the placebo (relative risk 1.18; 95% CI = 1.16 to 1.20). There was no statistical significance in the serious adverse events (relative risk 1.01; 95% CI = 0.95 to 1.07). A WHO-commissioned systematic review of serious adverse events (SAEs) showed no difference observed in the rates of selected SAEs between those exposed and those unexposed to the HPV vaccine [15,16]. 

The risk of anaphylaxis has been characterized as approximately 0.3–3 cases per million doses [16,17,18]. The global advisory committee on vaccine safety (GACVS) was presented data from the vaccine adverse event reporting system (VAERS) and the vaccine safety datalink (VSD) data with over 60 million doses administered, which showed no association identified between HPV vaccine and Guillain–Barré syndrome (Table 1). The recent studies included examination of specific outcomes that included complex regional pain syndrome (CRPS), Bell’s palsy, postural orthostatic tachycardia syndrome (POTS), premature ovarian insufficiency, primary ovarian failure, and venous thromboembolism, presenting no new evidence for a causal association between HPV vaccine and those conditions [16]. Although HPV vaccines have no link to cause adverse pregnancy outcome, it is not recommended in pregnancy. However, the safety profile in pregnant women on unintended administration demonstrated no safety concerns during the pre-licensure clinical trials or in post-licensure surveillance [19]. Additionally, new data from the VSD for > 92,000 eligible pregnancies were presented and no adverse obstetric, birth or structural abnormality outcomes were observed [16,20,21].

The nonavalent vaccine has more than double virus-like particles and aluminum adjuvant than the quadrivalent counterpart. As a consequence, the nonavalent vaccine had more frequent local and systemic adverse reactions than the quadrivalent vaccine (3.3% vs. 2.6%; *p*-value is 0.0125) [22]. Moreover, from the reporting rate to VAERS (after 259 reports per million nonavalent doses distributed) showed a serious report rate of 7 per million doses. In conclusion, there are no any new safety concerns from a group of prespecified adverse events in the nonavalent HPV vaccine. The safety profile of the nonavalent vaccine is similar to that of the quadrivalent HPV vaccine [23,24].

### 2.2. Immunogenicity

The pivotal studies have shown a remarkable essentially 99–100% seroconversion to all targeted HPV types in women 15–26 years of age. The immune response to the HPV vaccines is different from that to the natural infection, in which seroconversion is found in only 50–70% of HPV-infected women and 2–51% of males [32]. Long-term persistence of the immune response (8–9.4 year after three doses of the bivalent vaccine) significantly above natural infection levels was demonstrated (IgG level 10.8-fold and 10.0-fold higher than that after natural infection for HPV 16 and HPV 18, respectively). However, vaccine-induced antibody levels were higher in girls and boys than in young women [33,34].

A comparison of the immunogenicity between bivalent and quadrivalent vaccines in healthy women aged 18–45 years who received one dose or more regardless of the baseline HPV serostatus and the DNA status showed serum neutralizing antibody (nAb) responses induced by bivalent vaccine more than two times and six times higher than the levels observed with the quadrivalent vaccine for HPV subtypes 16 and 18, respectively (*p* < 0.0001). In addition, the bivalent vaccine resulted in nearly three times as many memory B cells for HPV subtypes 16 and 18 compared with the quadrivalent vaccine [35].

The seroconversion rate within 1 month after three doses of nonavalent vaccine was almost 100% for all nine HPV types and 77.5–100% of the participants remained seropositive after 5 years [36]. The antibody levels for HPV types 6/11/16/18 after the nonavalent vaccine were not different from those after the quadrivalent vaccine. In additional, the nonavalent vaccine was safe to give to individuals who had formerly received the HPV vaccine but desired to get protection against the five new HPV types. Similar to bi- and quadrivalent vaccines, a higher antibody response to nine HPV types was noted in young adolescents compared to young adults [37,38].

## 3. Efficacy and Effectiveness of the Human Papillomavirus Vaccine

The impact of HPV vaccination in real-world settings has become obvious, particularly among women who get vaccinated before HPV exposure in countries with high vaccine uptake. Maximal reductions of approximately 90% for HPV 6/11/16/18 infections, approximately 90% for genital warts, approximately 45% for low-grade cytological cervical abnormalities, and approximately 85% for high-grade histologically proven cervical abnormalities have been reported. The estimated vaccine effectiveness with one dose or more of the HPV vaccine was 83–96.1% [39,40].

### 3.1. Efficacy and Effectiveness of the HPV Vaccine in Young Women (under 26 Years Old)

The HPV vaccine is the most advantageous when given before the infection. For that reason, HPV vaccination is recommended for all 11- to 12-year-old. HPV vaccination is recommended to all people through 26 years as well if they did not get vaccinated when they were younger. The efficacy and effectiveness of different vaccine types in women under 26 years is summarized in Table 2.

The bivalent HPV vaccine (at least one dose) among young women who were previously uninfected showed a vaccine efficacy (VE) of 91–100% (95% CI = 64.6% to 86% and 94.2% to 100%, respectively) against HPV 16/18 incident and invoked significant cross protection against HPV types 31, 33, 35, 45, 53, and possibly 58. However, the efficacy against persistent infections with types 31 and 45 seemed to decrease with increased follow-up, suggesting a waning of cross protection [34,41,42,49]. In addition, the efficacy against HPV 16/18 infection irrespective of baseline HPV infection decreased to 76% (95% CI = 67% to 83%). In naive HPV infection before vaccination, the vaccine efficacy against the incidence of cervical intraepithelial neoplasia grade 2+ associated with HPV 16/18 was 92.9–97.4% (95% CI = 79.9% to 88.0% and 98.3% to 99.6%, respectively) and against cervical intraepithelial neoplasia grade 3+ was 87.0–94.9% (95% CI = 54.9% to 73.7% and 97.7% to 99.4%, respectively). The efficacy was lower irrespective of the baseline HPV infection. Moreover, the vaccine efficacy against CIN2+ irrespective of HPV DNA in lesions was 70.2% (95% CI = 54.7% to 80.9%) [40,44]. A positive impact of the bivalent HPV vaccine was observed, both on direct and cross protection.

Most vaccinations given in 2014 were the quadrivalent type, which targets oncogenic HPV types 6/11/16/18. The quadrivalent HPV vaccine shows excellent efficacy against genital warts in young women (pooled OR = 0.36; 95% CI = 0.26 to 0.51) and young male (pooled OR = 0.69; 95% CI = 0.61 to 0.78) [50]. The vaccine efficacy in an unrestricted susceptible population (USP) (negative polymerase chain reaction (PCR) and serologic testing at enrollment) was 100% (95% CI = 94% to100%) against the four HPV subtypes 3 years after vaccination. For CIN2+ and CIN3+ associated with HPV 6/11/16/18, the vaccine efficacy was 99% (95% CI = 91% to 100%) and 99% (95% CI = 82% to 100%). The quadrivalent demonstrated a cross-protection effect efficacy for HPV 31/33/45/52/58 of 46%, 29%, 7%, 18%, and 6%, respectively [15,43,45,49].

The nonavalent HPV vaccine became available in 2015, which targets the same types as the quadrivalent vaccine, plus five additional oncogenic types [40,51,52,53,54,55]. The vaccine efficacy in preventing persistent infections of HPV 31/33/45/52/58 ≥6 months after administration was 95.2% (95% CI = 81.4% to 98.4%) in naive HPV infection and 95.8% (95% CI = 87.8% to 98.9%) irrespective of the baseline HPV infection. For low- and high-grade disease associated with HPV 31/33/45/52/58, the vaccine efficacy in the per-protocol group was 97.6% (95% CI = 91.7% to 99.6%) and 96.7% (95% CI = 80.9% to 99.8%), while in the intention-to-treat group, the vaccine efficacy decreased to 84.0% (95% CI = 67.2% to 92.2%) in low-grade disease and 80.6% (95% CI = 33.7% to 94.3%) in high-grade disease [48]. A recent study reported that antibodies induced by the nonavalent vaccine could be transferred across the placenta, which potentially protects the infant from HPV 6 and 11 infections [39].

### 3.2. Efficacy and Effectiveness of the HPV Vaccine in Adult Women (>26 Years Old)

Although the HPV vaccine is approved for use in adults up to age 45, vaccination is not routinely recommended for persons older than 26 years because the advantage of the vaccine declines after exposure to HPV. However, there are several studies on vaccine effectiveness in adult women (Table 3).

The bivalent HPV vaccine efficacy against prevent persistent HPV 16/18 infection in adult, previously uninfected women was 83% (95% CI = 71% to 90%) and 43% (95% CI = 31% to 53%), respectively, irrespective of the baseline HPV infection. The vaccine efficacy against CIN2+ associated with HPV 16/18 was 70% (95% CI = 19% to 89%) in naive infection. The cross-protective vaccine efficacy against 6-month-persistent infection with HPV 31 was 79.1% (97.7% CI = 27.6% to 95.9%) and with HPV 45 was 76.9% (95% CI = 18.5% to 95.6%).

In the quadrivalent vaccine, even as the efficacy of the vaccine did not differ from that of the placebo against CIN2+ associated with HPV 6/11/16/182/3, the vaccine efficacy was 88.7% (95% CI = 78.1% to 94.8%) against CIN and external genital lesions related to HPV 6/11/16/18 in naive HPV infection at baseline compared with an efficacy of 30.9% (95% CI = 11.1% to 46.5%) in the ITT population [55,57]. The vaccine is low in effectiveness in patients with active HPV infection by vaccine HPV types or in patients with existing HPV-related lesions. However, in women with known previous exposure to a vaccine HPV type, but no active infection, the quadrivalent vaccine was shown to protect against reinfection or reactivation of the HPV type to which they had previously been exposed, as well as protecting against the other vaccine HPV types. In contrast, natural infection that had been cleared was not fully protective [58]. In women with previous or current infection with one or more vaccine HPV types, quadrivalent HPV vaccine provided protection against lesions caused by the remaining HPV types [59].

### 3.3. Efficacy and Effectiveness of the HPV Vaccine in Male

In males, of the HPV-attributable fractions of cancers, 92% of the anal cancer cases, 63% of the penile cancer cases, and 89% of the oral or oropharyngeal cancer cases are attributed to HPV types 16 and 18 [60,61].

The efficacy of the quadrivalent HPV vaccine in 10- to 15-year-old males was initially based on a (prelicensure) noninferiority immunobridging study by Block et al. The randomized, placebo-controlled, double-blind trial reported that the quadrivalent HPV vaccine reduced the incidence of external genital lesions related to HPV types 6, 11, 16, and 18 by 90% in 16- to 26-year-old males from 18 countries compared with the placebo and reported the efficacy in the intention-to-treat population as 65% (95% CI = 45% to 78%) [62,63].

The vaccine efficacy against the incident of HPV 16 and HPV 18 DNA detection was 28.0–45.1% and 33.9–49.5%, respectively. Vaccine efficacy estimates for preventing persisting (defined as ≥6 months) anogenital and anal infections were higher than those for incident infections (46.9–73.6%) [63,64]. Vaccine efficacy and effectiveness against anal condyloma was reportedly 57.2–67.2% [63,64]. Vaccine efficacy against AIN grade 1 was reported to be 49.6% and against AIN grade 2 was 61.9% [64], while vaccine effectiveness was slightly lower in a non-randomized study (50%). Efficacy against AIN grade 3 was reportedly a non-significant 46.8% [64]. In addition, PIN grade 2 or 3 was reported in one RCT, but the number of cases was too small in both the vaccinated (*n* = 3) and placebo groups (*n* = 2) to generate a meaningful estimate of vaccine efficacy [63].

In HPV DNA all negative at the study initial, the estimates of vaccine efficacy against persistent infection with HPV 6/11/16/18 was 68.3% and efficacy against DNA detection was 34.2%. Vaccine efficacy against the prevention of condyloma acuminata was higher than that among individuals irrespective of the HPV status, but the case numbers were small (10 cases) and did not lead to meaningful efficacy estimates [63]. The efficacy and effectiveness of HPV vaccination against human papillomavirus in males are reviewed in Table 4.

When vaccinating individuals, irrespective of their HPV status, vaccination is moderately effective against genital HPV infection and high-grade anal intraepithelial lesions. Higher vaccine efficacy was observed in those participants who were naive for the respective HPV types assessed in the individual studies. No meaningful estimate of vaccine efficacy could be calculated for high-grade penile intraepithelial lesions, and no data were available regarding vaccine efficacy or effectiveness against anal, penile, or head and neck squamous cell cancer.

## 4. The Real-World Effectiveness

Since the vaccine introduction, in 2006, the HPV vaccine has shown great impact in decreasing the prevalence of HPV type 6/11/16/18 infection in women aged 14–19 years old (prevalence decreased by 56%, 71%, and 88% in 4-, 8-, and 12-year-old, respectively). A study from the United States confirmed the statistically significant decline in the proportion of women infected with one or more of four valent vaccine-type HPV infections (80.9% decline), nine valent vaccine-type HPV infections (71% decline), and five valent vaccine-type HPV infections, apart from HPV 6/11/16/18 in nine valent vaccine-type infections (68.8% decline), among women who had received at least one dose of HPV vaccine. However, among the unvaccinated women, only the proportion infected with one or more of four valent vaccine-type HPV infections (40.1% decline) and five valent vaccine-type HPV infections, apart from HPV 6/11/16/18 in nine valent vaccine-type HPV infections (57.6% decline), was significant [65].

Declines in the prevalence and incidence of genital warts followed directly with decreases in HPV 6/11 infections, particularly in young women in high-vaccine-coverage countries. In women < 21 years of age, the reduction rate was 50% annually [66,67], whereas the reduction rate was lower in areas with a moderate to low coverage of vaccine [39]. Furthermore, reduction in four types of HPV infections and genital warts were observed in unvaccinated young men and women, consistent with herd protection.

Among the young women with three doses of vaccination, the CIN2+ and CIN3+ decline rates were 73–75% and 80–84%, respectively, compared with unvaccinated and partially vaccinated females; in contrast, in women aged between 20 and 29 years, the decline in CIN2+ and CIN3+ was 22% and 25%, respectively [68,69,70]. In Victoria, Australia, a similar age-related risk reduction was observed; among women of 12–26 years with at least one dose of vaccination, the decline in CIN2/CIN3/AIS ranged from 39% to 5% in younger and older groups, respectively, in comparison to unvaccinated women [71].

The incidence of cervical cancer was 6.7 per 100,000 among vaccinated women, compared with 11.3 per 100,000 among unvaccinated women. Among women vaccinated at age 16 years and younger, the incidence of cervical cancer remained low, at 0.01% with increasing age. However, in women vaccinated at 23–30 years and in unvaccinated women, the incidence increased abruptly at 23 years of age (when the screening program starts) and reached a maximum of 0.13% at age 30 years [7]. This corresponded with the incidence of cervical squamous cell carcinoma and adenocarcinoma, which demonstrated a higher average decrease rate in women aged 15–20 years (decrease, on average, of 12.7% and 4.1% per year, respectively) compared with those aged 25–29 years (decreased on average by 2.3% and 1.6% per year, respectively).

The most recent published study conducted in India showed that the vaccine efficacy against HPV 016 and 18 persistent infection was 95.4% (95% CI = 85.0% to 99.9%) in a single-dose cohort (Table 5) [5].

## 5. Alternative Schedules of the HPV Vaccine

Clinical studies evaluating reduced dose schedules and the intervals between doses for both vaccines have demonstrated non-inferior antibody responses in girls younger than 15 years of age who received two doses, given 6 months apart, when compared with women who received the standard three doses of vaccine and had evidence of efficacy in clinical trials. These findings have led to the recommendations and approval of two-dose schedules in 9- to 14-year-old girls [14,59]. Immunogenicity data for a single vaccine dose are limited. Although lower than the levels induced by two- and three-dose schedules, a single dose of HPV vaccine induced detectable HPV 16 and HPV 18 antibody levels higher than natural infection levels and remained 100% seropositive for 7 years [53,75].

Comparing three-dose, two-dose (0, 6 months), two-dose (0, 1 month), and one-dose groups, the cumulative incident HPV 16/18 infection rates after 7 years of vaccination were 4.3% (95% CI = 3.5% to 5.3%), 3.8% (95% CI = 1.0% to 10.1%), 3.6% (95% CI = 1.6% to 7.1%), and 1.5% (95% CI = 0.3% to 4.9%). The prevalence rates of other carcinogenic and noncarcinogenic HPV types, excluding HPV 16/18/31/33/45, were high and not statistically different among all dose groups, indicating that the low incidence of HPV 16/18 in the one- and two-dose groups was not due to a lack of exposure. The vaccine efficacy against prevalent HPV 16 or 18 infection was 80.2% (95% CI = 70.7% to 87.0%) among three-dose, 83.8% (95% CI = 19.5% to 99.2%) among two-dose, and 82.1% (95% CI = 40.2% to 97.0%) among single-dose women [53,76]. The incidence rate ratios compared with the unvaccinated for CIN2+ were 0.34 (95% CI = 0.13% to 0.87%), 0.49 (95% CI = 0.32% to 0.76%) and 0.43 (95% CI = 0.36% to 0.51%) after one, two, and three vaccine doses, respectively. The results were consistent for CIN3+. There was no difference in the incidence rates of CIN2+ and 3+ among women who had received three doses (CIN2+ 0.99, 95% CI = 0.64% to 1.53%; CIN3+ 0.95, 95% CI = 0.60% to 1.51%) or two doses (CIN2+ 1.00, 95% CI = 0.61% to 1.64%; CIN3+ 0.895, 95% CI = 0.53% to 1.52%) compared with women who had received one dose (Table 6) [77].

## 6. HPV Vaccine in Special Population

### 6.1. HIV Infection

Antibody responses were higher and showed a seroconversion rate close to 100% after vaccination with all HPV vaccines and no severe adverse events (RR = 0.6; 95% CI = 0.9 to 1.2) between vaccinated and placebo groups. However, the evidence on the clinical outcomes and harm of HPV vaccines in people with HIV needs further assessment of RCTs [54].

### 6.2. High-Risk Group: Men Who Have Sex with Men (MSM)

This group (MSM) had a high prevalence of high-risk HPV anal infection (41.2–53.6% of HIV-negative MSM and 65–85.1% of HIV-positive MSM). HPV 16 was the most common type (13.7% of HIV-negative MSM and 28.5% of HIV-positive MSM). A higher number of male sex partners in a lifetime was significantly associated with anal and penile high-risk HPV in HIV-negative MSM [78,79,80].

Prevalence rates of any HPV types were identified as 17.1% (95% CI = 7.3–26.8%) and 28.9% (95% CI = 19.1–38.7%) of oral samples from HIV-positive and HIV-negative MSM. Similarly, HPV 16 was the most frequently detected type: (3.0% (95% CI = 0.5–5.5%) in HIV-negative and 4.7% (95% CI = 2.1–7.3%) in HIV-positive MSM). Oral infection with high-risk HPV was statistically significant associated with HIV infection [81,82].

Although many individuals in the MSM population have already been infected with HPV and may benefit from natural immunity, there is some evidence that a previous infection with one type of HPV does not necessarily protect against a new infection (or reinfection/reactivation) with the same type of HPV. Only 4–36% of the men develop detectable antibodies after a recent infection with HPV [83]. The herd effect from the female vaccination program is useful in heterosexual men, but there are no real benefits in the MSM group [84].

The efficacy of the quadrivalent vaccine against external genital lesions in MSM was 79.0% (incidence of 3.7 per 100 person-years in the vaccinated group compared with 7.3 per 100 person-years in the unvaccinated group; *p* = 0.05) [63,85]. Moreover, the vaccine was associated with a decreased risk of recurrent high-grade anal intraepithelial neoplasia (HR 0.50; 95% CI = 0.26–0.98; *p* = 0.04) [86]. Meanwhile, the nonavalent vaccination showed anti-HPV seroconversion 96.4–100% (95% CI = 96.6–100% to 98.4–100%) in all nine types in MSM.

However, the quadrivalent HPV vaccination showed that the antibody responses to all four vaccine types were lower in MSM than in heterosexual men after 7 months of vaccination, with GMT ratios (MSM/HM) ranging numerically from 0.48 to 0.66; this is similar to the responses with the nonavalent HPV vaccine (GMT ratios at month 7 of nine HPV types (MSM/HM) ranged from 0.59 to 0.75) [87,88].

In recognition of the elevated anal cancer risk and possible absence of herd immunity among MSM, some countries recommend targeted HPV vaccination of MSM, such as the national human papillomavirus (HPV) vaccination programme for gay, bisexual and other men who have sex with men (MSM) in the UK, which has been offering vaccination to MSM aged up to 45 years since April 2018 [89].

## 7. Effectiveness of the HPV Vaccine on Other Cancers

The most common two HPV types, HPV 16 and 18, are associated with head and neck cancers, including oral squamous cell carcinoma, oropharyngeal squamous cell carcinoma, and laryngeal squamous cell carcinoma. HPV-positive oropharyngeal cancer presents in a younger, healthier population with a unique set of risk factors and a good prognosis for survival [90]. The prevention of oral infection is due to the presence of salivary antibodies that follow seroconversion and correlated with the serum level. Anti-HPV 16 antibodies in the oral cavity were detected in 96% and anti-HPV 18 were detected 72% of the mouthwash specimens [91,92]. HPV vaccine effectiveness up to 6 years post vaccination was 82.4% (95% CI = 47.3% to 94.1%) on HPV 16/18, 75.3% (95% CI = 12.7% to 93.0%) on HPV 31/45, 69.9% (95% CI = 29.6% to 87.1%) on HPV 31/33/45, and 25.8% (95% CI = −21.7% to 54.8%) on the low-risk HPV 6/11. No vaccine effectiveness was found against other high-risk HPV types. The relative reduction in HPV 16/18 was 82.4% [93]. Vaccinated adults had a lower prevalence of oral HPV types 6, 11, 16, and 18 compared to unvaccinated adults. (0.11% vs. 1.61%; *p* (adj) = 0.008); the prevalence of non-vaccine high-risk oral HPV was similar between HPV-vaccinated and unvaccinated participants [94,95].

HPV 16 is the most carcinogenic HPV type in the anus, both in HIV-negative and HIV-positive individuals of both sexes. HPV 16 positivity is enhanced with the severity of the anal diseases. However, HPV-16-positive cancers were less frequent in HIV-positive compared with HIV-negative patients (67.1% vs. 85.5%) [96]. The HPV vaccine has been demonstrated to reduce persistent anal infection with HPV 6, 11, 16, or 18 by 59.4% (95% CI = 43.0% to 71.4%) and 94.9% (95% CI = 80.4% to 99.4%) in the intention-to-treat population and the per-protocol population, respectively. The efficacy of the HPV vaccine against anal intraepithelial neoplasia associated with HPV 6, 11, 16, or 18 was 50.3% (95% CI = 25.7% to 67.2%) in the intention-to-treat population and 77.5% (95% CI = 39.6% to 93.3%) in the per-protocol efficacy population; the efficacies against anal intraepithelial neoplasia associated with HPV of any type were 25.7% (95% CI = −1.1% to 45.6%) and 54.9% (95% CI = 8.4% to 79.1%). The reduction rate of grade 2 or 3 anal intraepithelial neoplasia related to infection with HPV 6, 11, 16, or 18 was 54.2% (95% CI = 18.0% to 75.3%) in the intention-to-treat population and 74.9% (95% CI = 8.8% to 95.4%) in the per-protocol efficacy population [64]. Moreover, the HPV vaccine significantly decreased the rate of recurrent AIN2+ in HIV-negative MSM [86].

HPV infection can cause a variety of cutaneous manifestations, including (i) common warts caused by HPV 2, HPV 7, HPV 27, and HPV 57 (Alpha genus), (ii) filiform warts caused by HPV 4 and HPV 60 (Mu genus), (iii) palmar and plantar warts caused by HPV 1 (Nu genus), and (iv) HPV 5 and 8 (Beta genus) associated lesions; these include epidermodysplasia verruciformis (EV), an autosomal recessive disorder with mutations in EVER1 or EVER2 genes on chromosome 17q25 (1), and non-melanoma skin cancer (NMSC) that includes basal cell carcinoma (BCC) and squamous cell carcinoma (SCC) [97].

Although the main risk factors for NMSC are UV radiation, genetics, and immunosuppression, Beta-HPVs may also play a role in the NMSC pathogenesis. Beta-HPV DNA has been found in up to 65% of cutaneous SCC tumors [98,99] and in up to 50% of BCC tumors [100]. These viruses can also be found in premalignant lesions, such as actinic keratosis [101,102]. Additionally, Beta-papillomaviruses DNA is more common in lesions from an immunocompromised host (90%) [103].

The evidence that virus-like-particle (VLP)-based vaccines induce effective neutralizing antibodies against cutaneous papillomaviruses and prevent skin tumors in immunocompetent and immunocompromised conditions derive from the preclinical setting [104].

A few studies have focused on the use of an L2-derived vaccine to enhance the protection against cutaneous papillomaviruses. Antibodies derived from HPV-16-derived L2 peptide can cross neutralize in vitro against several types of HPV, including HPV 2, 3, 5, 8, 23, 27, 38, 57, and 76. The cross neutralization can also be achieved in vivo by cutaneous challenge with pseudo-virions, highlighting the potential of the L2-derived vaccines to confer protection against a broad range of cutaneous HPVs. However, the effectivity of such vaccines in preventing skin tumors has not been assessed so far [105,106,107].

## 8. Therapeutic Vaccine

The aim of a therapeutic vaccine against HPV is to induce in vivo virus-specific T-cell responses against established HPV infections and lesions. For therapeutic vaccination, it is important to maximize the T-cell responses that induce optimal effector profiles and reach the tumor-specific size [108]. In brief, most therapeutic vaccines have used E6 or E7 or a combination of both as a target antigen. Since HPV oncoproteins E6 and E7 are expressed only at the tumor cells, which makes them ideal targets for therapeutic vaccines. Therapeutic vaccines have been developed on a wide variety of platforms and include peptide- or protein-based vaccines, viral vector vaccines, bacterial vector vaccines, cell-based vaccines, DNA-based vaccines, and RNA-based vaccines [109,110].

To date, there are no therapeutic vaccines that have irreversibly cured HPV-associated cervical cancers. However, there are a few promising therapeutic vaccine candidates, including the HPV type 16 E-7 expressing Lactobacillus-based vaccine for the treatment of HPV-16-vaccine-positive HSIL [111] and the VGX-3100 DNA vaccine with electroporation for patients with cervical intraepithelial neoplasia (CINI) grade 2/3 or 3. The results of the latter study showed regression of the lesions to CINI and clearance of HPV 16/18, helped avoid excision at 6 months following treatment completion, and had no detectable HPV 16/18 at 18 months following treatment completion [112]. There are also a few clinical trials combining therapeutic vaccines with antibodies against programmed death-ligand 1 (PD-L1) and that have been reported to increase immune responses leading to the suppression of tumor growth [113]. A wide variety of cell-based vaccines have some limitations. RNA-viral-based vectors have also been explored. Only one RNA viral vector, Semliki Forest virus (SFV) replicons encoding E6 and E7, known as Vvax001, is entering a Phase I trial for safety and efficacy in humans [114]. Protein-based vaccines are processed by antigen presenting cells (APCs) with potential advantages of safety and tolerability and specially for immunocompromised individuals. One of the most advanced protein-based vaccines SGN00101 (also known as HPS E-7) is based on the fusion of HPV 16 E7 with recombinant heat shock protein 65 (HSP65) from Mycobacterium bovis [115].

## 9. Conclusions

All vaccines presented exceptional protection against HPV infection, cervical intraepithelial neoplasia of grade 2 or 3 (CIN2 or CIN3), and adenocarcinoma in situ associated with HPV 16/18 infection. The efficacy and effectiveness of vaccines were noticeably high among young women who were HPV seronegative before vaccination. Vaccine efficacy was lower when women regardless of HPV DNA status at enrollment were included.

Vaccine efficacy and effectiveness were lower in adult women (aged 26–45 years), especially protection against CIN2/3 associated with HPV 16/18 and against persistent HPV 16/18 infection. The reduction is significant in the intention-to-treat group, while there are no differences in younger and adult women in the per-protocol group that was HPV 16/18 negative at baseline [15]. Moreover, in adult women, it is possible that they have been vaccinated for reasons related to a higher risk of cervical cancer because of sexual behavior and lifestyle and other health factors. Another potential explanation is that these women have a much higher likelihood of already being exposed to HPV before vaccination.

A comparison of bivalent, quadrivalent, and nonavalent efficacy against HPV 16/18 showed it to be similar [15,48,116]. However, the nonavalent vaccine can bestow more advantages by increasing coverage to HPV 31/33/45/52/58. The expansion of vaccine coverage to HPV 52 and 58 is particularly important in Asia due to the relatively high prevalence of these types [46]. Better cross protection against HPV 31/33/45 was shown by the bivalent vaccine. Though there are some suggestions of diminishing cross protection [49], the efficacy of the bivalent vaccine against HPV 31/33/45 infection continuing for more than 9 years has been described [116].

In real-world settings, rapid reductions were first demonstrated in young women with high coverage of HPV vaccine. The significant decline in HPV 6/11/16/18 in vaccinated women compared with unvaccinated women showed the high effectiveness of the vaccine. Nevertheless, the reduction in HPV 6/11/16/18/31/33/45/52/58 results from direct protection by nonavalent HPV vaccine and the cross protection provided by bivalent and quadrivalent vaccine. In addition, there was a significant decline in HPV 6/11/16/18 in unvaccinated women, deriving from herd protection. Evidence about herd protection will be a key component of cost-effectiveness analysis.

A two-dose HPV vaccination schedule is simpler and less expensive than a three-dose schedule. Immunogenic data showed non-inferior results in a two-dose compared with a three-dose schedule of any HPV vaccines. There is no difference in seroconversion between two-dose and three-dose schedules at all time points reported; almost all participants seroconverted in both groups. The use of a single-dose HPV vaccination schedule remains controversial. Full results will emerge from some large studies next year. In the meantime, a single-dose schedule may be feasible for the hard-to-reach population.

For males, the quadrivalent HPV vaccine possibly reduces the incidence of external genital, lesions including condyloma accuminata, AIN grades 1 and 2, and persistent infection by HPV 6/11/16/18. Limited data were available regarding the efficacy and adverse events with bivalent vaccines in males.

In people living with HIV, even if the antibody response with all HPV vaccines showed a seroconversion rate close to 100% in HIV-infected people, evidence about the efficacy and harm of HPV vaccines is limited. The duration of protection of HPV vaccines in people with HIV infection and the effect of declining immunity on protection are unknown.

The prevalence of high-risk HPV infection and the related diseases was high in men who have sex with men (MSM). In addition, there were evidences of herd protection in some communities thus HPV vaccine is indicated in this group in some countries.

HPV vaccination showed high effectiveness against oral HPV type 16/18 infection, and a significant percentage of participants developed IgG antibodies in oral fluid post vaccination. However, low prevalence of HPV infection in the asymptomatic population, low vaccine uptake rate, and long duration between infection and cancer development result in the vaccine effectiveness reducing the incidence of and mortality related to HPV-related head and neck cancer, which should be observed long term. However, the FDA recently approved oropharyngeal cancer preventions as an indication for HPV vaccines [117].

Because of the excellent vaccine efficacy against HPV anal infection and anal intraepithelial neoplasia, the Vaccines and Related Biological Products Advisory Committee (VRBPAC) of the FDA recommends approval of the quadrivalent and nonavalent HPV vaccines for the prevention of anal cancer linked to HPV types 16, 18, 6, and 11 in males and females from 9 to 26 years of age [117,118].

Therapeutic vaccine against HPV proposes to induce in vivo virus-specific T-cell responses against established HPV infections and lesions. Even though there are no therapeutic vaccines that can irreversibly cure HPV-associated cervical cancers up to the present, there are a few suggested therapeutic vaccine candidates for example: the VGX-3100 DNA vaccine, the HPV E6/E7 Peptide vaccine, the Vvax001 RNA vaccine, and the SGN00101 protein-based vaccine.

Although HPV vaccines have very high effectiveness, women who have received the HPV vaccine series should still be screened for cervical cancer, beginning at age 21, in accordance with current cervical cancer screening guidelines.

## Figures and Tables

**Table 1:** Adverse effects of bivalent and quadrivalent HPV vaccines.

Adverse Effect	Vaccine Type	Relative Risk	95% CI
Overall adverse effects at the injection site [15]	Bivalent and quadrivalent	1.18	1.16 to 1.20
Overall systemic events [15]	Bivalent and quadrivalent	1.02	0.98 to 1.07
Serious adverse event [15]	Bivalent And quadrivalent	1.01	0.95 to 1.07
Autoimmune-related conditions [25]	Bivalent	0.98	0.80 to 1.21
Thromboembolic event [26]	Quadrivalent	0.7	0.3 to 1.4
Chronic fatigue syndrome [27]	Quadrivalent	0.94	0.78 to 1.14
Multiple sclerosis [28]	Quadrivalent	0.3	0.1 to 0.9
Connective disorders [28]	Quadrivalent	0.8	0.3 to 2.4
Type 1 diabetes [28]	Quadrivalent	1.2	0.4 to 3.6
Guillain–Barré syndrome (GBS) [29]	Bivalent and quadrivalent	3.78	1.79 to 7.98
	Bivalent	8.08	1.69 to 38.61
	Quadrivalent	3.78	1.70 to 8.41
Thyroiditis [30]	Bivalent	3.75	1.25 to 11.31
Inflammatory bowel disease [29]	Bivalent and quadrivalent	1.14	0.97 to 1.35
	Bivalent [31]	1.11	0.75 to 1.66

**Table 2:** Vaccine efficacy and effectiveness (at least one dose) in women under 26 years old.

Efficacy and Effectiveness	Vaccine Type	HPV Status at Enrolment	Vaccine Efficacy (95% CI)
Efficacy against HPV 16/18 infection [15,34,41,42]	Bivalent	Naive	91–100% (64.6% to 86%; 94.2% to 100)
		Irrespective	76% (67% to 83%)
Persistent infection with HPV 16/18 (6 months) [15]		Naive	90% (87% to 92%)
		Irrespective	56% (49% to 62%)
Persistent infection with HPV 16/18 (12 months) [43]		Irrespective	97.7% (83.5% to 99.7%)
Persistent infection with HPV 31/33/45 (12 months) [43]		Irrespective	61.8% (16.7% to 82.5%)
CIN2+ associated with HPV 16/18 [15,40,44]		Naive	92.9–97.4% (79.9% to 88.0%; 98.3% to 99.6)
		Irrespective	54% (43% to 63%)
CIN3+ associated with HPV 16/18 [15,40,44]		Naive	87.0–94.9% (54.9% to 73.7%; 97.7% to 99.4%)
		Irrespective	74% (55% to 91%)
Any CIN2+ irrespective of HPV type [40]		Naive	70.2% (54.7% to 80.9%)
Any CIN3+ irrespective of HPV type [15]		Naive	92% (77% to 97%)
		Irrespective	45% (29% to 57%)
Efficacy against external anogenital and vaginal lesions associated with HPV 6/11/16/18 [45]	Quadrivalent	Naive	100% (94% to 100%)
Persistent infection with HPV 6/11/16/18 (6 months) [15]		Naive	87% (63% to 95%)
CIN2+ associated with HPV 6/11/16/18 [15]		Naive	99% (91% to 100%)
		Irrespective	50% (41% to 58%)
CIN3+ associated with HPV 6/11/16/18 [15]		Naive	99% (82% to 100%)
Any CIN2+ irrespective of HPV type [15]		Naive	43% (24% to 56%)
Any CIN3+ irrespective of HPV type [15]		Naive	46% (17% to 64%)
		Irrespective	19% (4% to 31%)
Persistent infection with HPV 31/33/45/52/58 (≥6 months) [46,47]	Nonavalent	Naive (3 doses)	95.2% (92.7% to 97.0%)
		Irrespective	95.8% (87.8% to 98.9%)
Persistent infection with HPV 31/33/45/52/58 (≥12 months) [46,48]		Naive (3 doses)	96.3% (94.4% to 97.7%)
		Irrespective	93.9% (81.4% to 98.4%)
CIN2/3, adenoma in situ, and cervical cancer associated with HPV 31/33/45/52/58 [47]		Naive (3 doses)	90.9% (46.4% to 99.6%)
Low-grade disease associated with HPV 31/33/45/52/58, including condyloma, CIN1, vulvar intraepithelial neoplasia 1, and vaginal intraepithelial neoplasia 1 [48]		Naive (3 doses)	97.6% (91.7% to 99.6%)
		Irrespective	84.0% (67.2% to 92.2%)
High-grade disease associated with HPV 31/33/45/52/58, including CIN2/3, adenoma in situ, cervical cancer, vulvar intraepithelial neoplasia 2/3, vulvar cancer, vaginal intraepithelial neoplasia 2/3, and vaginal cancer [48]		Naive (3 doses)	96.7% (80.9% to 99.8%)
		Irrespective	80.6% (33.7% to 94.3%)

**Table 3:** Vaccine efficacy and effectiveness (at least one dose) in adult women (>26 years old).

Vaccine Type	Efficacy and Effectiveness	HPV Status at Enrolment	Vaccine Efficacy (95% CI)
Bivalent	Persistent infection from HPV 16/18 (6 months) [15,56]	Naive	83% (71% to 90%)
		Irrespective	43% (31% to 53%)
	CIN2+ associated with HPV 16/18 [15]	Naive	70% (19% to 89%)
		Irrespective	26% (−5% to 48%)
Quadrivalent	Persistent infection from HPV 6/11/16/18 (6 months) [15]	Irrespective	48% (35% to 58%)
	CIN2+ associated with HPV 6/11/16/18 [15]	Naive	63% (−41% to 90%)
		Irrespective	22% (−37% to 56%)
	All CIN and external genital lesions related to HPV 6/11/16/18 [57]	Naive	88.7% (71.8% to 94.8%)
		Irrespective	30.9% (11.1% to 46.5%)
	Incidence of infection of at least 6 months’ duration and cervical and external genital disease related to HPV 6/11/16/18 [57]	Naive	74·6% (58.1% to 85%)
		Irrespective	30.9% (11.1% to 46.5%)

**Table 4:** Efficacy and effectiveness of HPV vaccination against human papillomavirus in males.

Outcome	Type of Vaccine	Dose of Vaccine	HPV Status at Enrolment	Vaccine Efficacy or Effectiveness (95% CI)
Seroconversion after 1 month to HPV 6/11/16/18 [63]	Quadrivalent vaccine	Three doses	Irrespective	97.4%
DNA detection of HPV (intention-to-treat population) [63,64]	Quadrivalent vaccine	At least one dose		
HPV 6			Irrespective	35.1% (20.3% to 47.3%) to 61.5% (42.3% to 74.8%)
			Naive	46.5% (30.2% to 59.2%)
HPV 11			Irrespective	43.2% (18.7% to 60.7%) to 54.7% (22.6% to 74.3%)
			Naive	50.5% (20.1% to 70.0%)
HPV 16			Irrespective	28.0 (12.9 to40.7) to 45.1% (18.0% to 63.7%)
			Naive	29.4% (10.1% to 44.7%)
HPV 18			Irrespective	33.9% (13.0% to 50.1%) to 49.5% (11.3% to 72.1%)
			Naive	45.0% (23.7% to 60.7%)
Persistent infection (intention-to-treat population) [63,64]	Quadrivalent vaccine	At least one dose		
HPV 6			Irrespective	44.7% (24.1 to 60.1) to 62.5%(37.5 to 78.2)
HPV 11			Irrespective	53.7% (7.5 to 78.0) to 59.4%(25.7 to 78.8)
HPV 16			Irrespective	46.9% (28.6 to 60.8) to 54.0%(23.9 to 72.9)
HPV 18			Irrespective	56.0% (28.2 to 73.7) to 73.6%(37.5 to 90.3)
Condyloma acuminate [63,64]	Quadrivalent vaccine	At least one dose	Irrespective	57.2(15.9 to79.5) to 67.2% (47.3% to 80.3%)
PIN grade 1 [63]	Quadrivalent vaccine	At least one dose	Irrespective	25.6% (−339.9 to 89.1)
PIN grade 2 or 3 [63]	Quadrivalent vaccine	At least one dose	Irrespective	−48.9% (−1682.6 to 82.9)
AIN grade 1 [64]	Quadrivalent vaccine	At least one dose	Irrespective	49.6% (21.2% to 68.4%)
AIN grade 2	Quadrivalent vaccine	At least one dose	Irrespective	61.9% (21.4% to 82.8%)
AIN grade 3	Quadrivalent vaccine	At least one dose	Irrespective	46.8% (−20.2% to 77.9%)

**Table 5:** Impact of HPV vaccine in decreasing prevalence and incidence of HPV infection and cervical cancer.

Outcome	Dose of Vaccine	Population Group	Duration after Vaccination	Result
Prevalence of infections of HPV types 6, 11, 16, and 18 [52,65,72]	At least one dose	14–19 years old	4 years	Decreased 56%
			8 years	Decreased 71%
			12 years	Decreased 88%
Prevalence of HPV 6/11/16/18/31/33/45/52/58 infections [65]	At least one dose	14–19 years old	12 years	Decreased 65%
Incidence of cervical squamous cell carcinoma (SCC) [73]	At least one dose	15–20 years old	18 years	Decreased on average by 12.7% per year
		25–29 years old	18 years	Decreased on average by 2.3% per year
Incidence of adenocarcinoma [73]	At least one dose	15–20 years old	18 years	Decreased on average by 4.1 per year
		25–59 years old	18 years	Decreased on average by 1.6 per year
Vaccine efficacy against persistent HPV 16 and 18 infections [74]	Single dose	10–18 years old	9 years	Vaccine efficacy 95.4% (95% CI = 85.0% to 99.9%)
	Two doses	10–18 years old	9 years	Vaccine efficacy 93.1% (95% CI = 77.3% to 99.8%)
	Three doses	10–18 years old	9 years	Vaccine efficacy 93.3% (95% CI = 77.5% to 99.7%)

**Table 6:** Impact of the number of administrated doses.

Outcome	3 Doses (95% CI)	2 Doses (95% CI)	1 Dose (95% CI)
Incident of HPV 16/18 infection [53]	4.3% (3.5% to 5.3%)	0, 6 months; 3.8% (1.0% to 10.1%) 0, 1 month; 3.6% (1.6% to 7.1%)	3.6% (0.3% to 4.9%)
Vaccine efficacy against prevalence of HPV 16/18 [76]	80.2% (70.7% to 87.0%)	83.8% (19.5% to 99.2%)	82.1% (40.2% to 97.0%)
Relative risk of 6 months persistent HPV 16/18 infection in women (naive HPV infection) [15]	0.067 (0.049 to 0.093)	0.126 (0.126 to 0.544)	0.045 (0.003 to 0.774)
Incidence rate ratios for cervical intraepithelial neoplasia grade 2 compared with unvaccinated women [77]	0.43 (0.36 to 0.51)	0.49 (0.32 to 0.76)	0.34 (0.13 to 0.87)
Incidence rate ratios for cervical intraepithelial neoplasia grade 3 compared with unvaccinated women [77]	0.37 (0.30 to 0.45)	0.38 (0.22 to 0.66)	0.38 (0.14 to 0.98)
Incidence rate ratios for cervical intraepithelial neoplasia grade 2; comparison of the number of doses administered among vaccinated women [77]	0.99	1.00	1
	(0.64 to 1.53)	(0.61 to 1.64)	
Incidence rate ratios for cervical intraepithelial neoplasia grade 3; comparison of the number of doses administered among vaccinated women [77]	0.95	0.89	1
	(0.60 to 1.51)	(0.53 to 1.52)	
